# The Drivers of Heuristic Optimization in Insect Object Manufacture and Use

**DOI:** 10.3389/fpsyg.2018.01015

**Published:** 2018-06-21

**Authors:** Natasha Mhatre, Daniel Robert

**Affiliations:** ^1^Department of Biological Sciences, University of Toronto at Scarborough, Scarborough, ON, Canada; ^2^School of Biological Sciences, University of Bristol, Bristol, United Kingdom

**Keywords:** insect cognition, object manufacture, baffling behavior, objective function, optimization, heuristics

## Abstract

Insects have small brains and heuristics or ‘rules of thumb’ are proposed here to be a good model for how insects optimize the objects they make and use. Generally, heuristics are thought to increase the speed of decision making by reducing the computational resources needed for making decisions. By corollary, heuristic decisions are also deemed to impose a compromise in decision accuracy. Using examples from object optimization behavior in insects, we will argue that heuristics do not inevitably imply a lower computational burden or lower decision accuracy. We also show that heuristic optimization may be driven by certain features of the optimization problem itself: the properties of the object being optimized, the biology of the insect, and the properties of the function being optimized. We also delineate the structural conditions under which heuristic optimization may achieve accuracy equivalent to or better than more fine-grained and onerous optimization methods.

*“It is demonstrable,” said he*,

“that things cannot be otherwise than they are;

*for as all things have been created for some end*,

they must necessarily be created for the best end.”

Candide, or Optimism – Voltaire

## Object Manufacture by Insects

Animals make and use a large variety of objects for a range of functions, mainly constructions that they inhabit or use as traps, tools that they use for food acquisition or for increasing their reach, or objects they use to create displays that attract mates or warn rivals (Hansell, 2007). Interestingly, insects seem to participate in the full gamut of object use and manufacture despite their small body- and brain size. Indeed, it is likely that object manufacture is more prevalent in insects than in non-human vertebrates (Hansell, 2007). For instance, insects make a variety of intricate nests and inhabitations that provide protection and even climate control (Korb, 2007). Ant-lions build pitfall traps to capture ground dwelling prey (Devetak et al., 2005; Fertin and Casas, 2006), ants drop stones and soil using them as projectile weapons as they raid a bee’s nest for pollen (Lin, 1964; Schultz, 1982). Other insects like caddis-fly, lacewing and reduviid larvae defend or camouflage themselves by covering themselves with debris (Livingstone and Ambrose, 1986; Ferry et al., 2013; Tauber and Tauber, 2014). Crickets manufacture acoustic objects like baffles and burrows that help them increase the loudness of their mating calls (Bennet-Clark, 1987; Mhatre et al., 2017). More uniquely, insects are the only animals known to make objects out of their own bodies, objects that have been dubbed self-assemblages (Anderson et al., 2002). For example, ants make bridges that help them travel more efficiently over gaps in the substrate (Reid et al., 2015; Graham et al., 2017), others make rafts that allow them to float and survive a flood (Mlot et al., 2011). Bees are even known to make predator killing ovens using their own body heat (Ken et al., 2005).

Many of these objects have to be made in variable environmental contexts without fixed properties. Thus, optimal or even functional manufacture may not be possible using a stereotyped behavioral program. It is proposed that manufacture behavior needs to be responsive to features of the environmental demands that constitute the problem. For instance, some objects must function in different weather: termite mound architecture has to respond to local climatic conditions. In West Africa, shrub savanna conditions are warm but thermally unstable, and termite mounds with many ridges and turrets function better, whereas compact mounds with a dome-like structure perform better in the cooler but more stable gallery forests (Korb and Linsenmair, 1998). Thus, it can be hypothesized that a behavioral program that flexibly responds to local climatic conditions that the termites find themselves in, would be better than a stereotypical, “one size fits all” behavior. Another problem feature that calls for flexibility is material efficiency: the range of materials that can be used to manufacture the objects, each of which may have different efficiencies, requires animals to be able to select intelligently within the range available to them. Material efficiency is understood here as the collection of material properties that confer functionality in specific uses, e.g., hardness, size, weight, color, insulation, biodegradability, wettability, thermal mass, to name a few. For example, tree crickets can choose from a wider variety of leaf sizes to make baffles, and this distribution changes as the seasons and hence plant phenology progresses. However, only a narrow range of the available leaves make an optimal or ‘worthwhile’ baffle, which the crickets must choose from while also balancing search times (Mhatre et al., 2017). In still other cases, the problem itself may be variable and may necessitate a flexible behavioral program. For example, the size of the gap in the environment over which army ants must build a bridge is dependent on the environment itself, and hence highly variable. The ants must follow a behavioral program that enables flexible decision making that balances the cost of ants used to make the bridge against the travel distance saved (Reid et al., 2015; Graham et al., 2017). In view of this complexity, establishing whether and how optimal solutions are reached in insects remains a challenging task.

It was commonly believed that insects have simple and highly stereotypical behavior as a result of a small brain size. Insect behavior was proposed to follow stereotypical and rather inflexible behaviors, the so-called fixed action patterns, in response to a particular set of sensory inputs (Gould and Gould, 1982). This description of insect behavior is at odds with the evidence and need for flexibility in object manufacture described in more recent literature. Indeed, it is also at odds with the proposition that insects optimize the objects they manufacture. More recent work, in effect, has challenged the notion that larger brains are better at cognitive tasks (Chittka and Niven, 2009). Small brained insects have been demonstrated to exhibit remarkably sophisticated cognitive behavior, such as numerosity (Dacke and Srinivasan, 2008) and concept abstraction (Giurfa et al., 2001; Avarguès-Weber et al., 2012). Using a ‘constructive’ approach, researchers have found that even relatively small neural networks can produce similarly sophisticated behavior (Dehaene et al., 1987; Dehaene and Changeux, 1993; Beer, 2003). The idea that even minimal computational programs can enable flexible, responsive and hence intelligent behavior is increasingly gaining traction. In this review, we consider the different kinds of minimal computational procedures that might be used by insects to optimize the objects they manufacture.

## Optimization: Definitions and Criticisms

**FIGURE 1:**
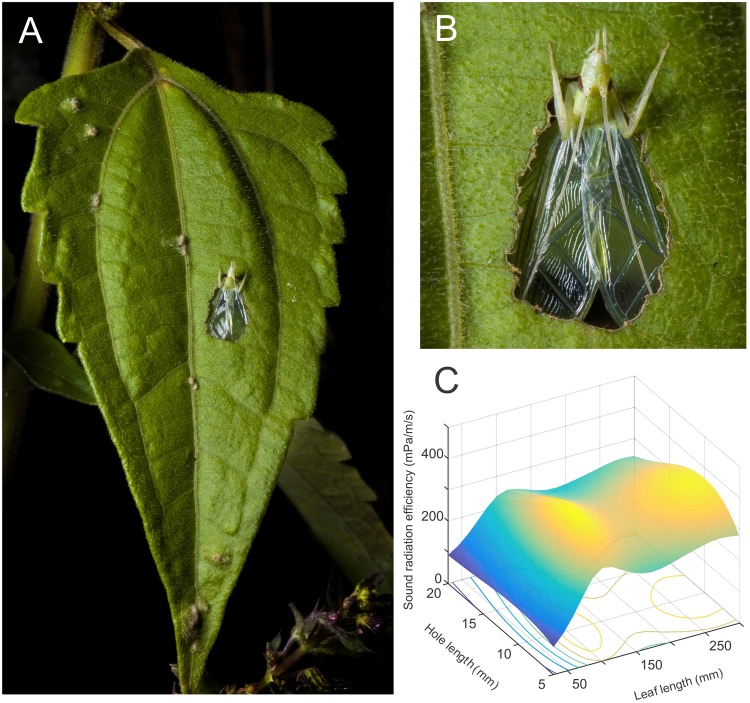
A baffle as an example of an object optimization problem. **(A)** Tree cricket males cut a hole in a leaf to create an object called a baffle. **(B)** This baffle is used by the cricket during singing and the male places its wings against the hole and parallel to the leaf surface while producing sound from its vibrating wings. **(C)** This device improves the crickets sound radiation efficiency. This efficiency is controlled by two main parameters, the size of the leaf they use and the size of the hole (reproduced from data in Mhatre et al., 2017). To optimize a baffle, the animal must find the highest point on this objective function. This is a 2 dimensional objective function. Objective functions, in general, however, may be determined by any number of parameters and each parameter will correspond to one dimension of the function. A baffle can in principle have any combination of positive leaf length and hole length and this would form the domain of the problem. Realistically, however, the leaf sizes available to the insect depend on naturally available leaves (∼11–141 mm) and the hole sizes depend in turn on leaf size. This smaller subspace is called the feasible region of the objective function.

In formal mathematical terms, optimization is a process by which we find the maximum or minimum value of some function (**Figure 1C**). This function is called the objective function, and the input space over which this function varies is defined as the domain. Real world optimization problems tend to have a feasible domain, i.e., the space defined by the subset of inputs that can be realistically achieved (**Figure 1**). Such a simple definition, admittedly, hides the wide range of problem types and approaches encompassed in optimization theory (Foulds, 1948).

Optimization in a biological context poses different problems. It is generally understood that in most cases optimization behavior is inherited and the animal is not intentionally seeking the optimum of the objective function. It is expected that the animal has a preference for some quantity which it maximizes without intention. During this behavior, the animal does not know, or have an expectation of, how the objective function will change as it performs modifications to its behavior and it discovers this function via a search. Additionally, the suite of behaviors that are regarded to be under an optimization process can only arise through an evolutionary process, which itself involves interactions with the environment and other organisms. Unsurprisingly, as these interactions are dynamic and involve co-evolutionary processes, such as arms-races, objective functions may be intrinsically dynamic and present varying optimal points (McFarland, 1977; Smith, 1978; Parker and Smith, 1990).

In this fluid context, the most serious challenge to the contention that animals can optimize is that it is an overly optimistic or ‘Panglossian’ outlook (Gould and Lewontin, 1979). Taking a page from Voltaire’s play, Candide, Gould and Lewontin (1979) in their seminal paper argued against a then-prevalent tendency to view biological traits as unitary, and as having been optimized by evolutionary forces. They argued that it was more realistic to view traits as part of larger *Baupläne* and therefore constrained by phylogeny, development and physical and architectural constraints. The authors argued that this means that most traits are not likely to be functionally ‘optimal’ (Gould and Lewontin, 1979). Indeed, the idea that a biological trait develops for a single unambiguous function contains a teleological argument and is therefore problematic (Pierce and Ollason, 1987). Different authors have dealt with these criticisms. The chief response does not resolve the issues raised but argues instead that optimization can be a productive hypothesis; one that then enables insights into the cost-benefit trade-offs and the phylogenetic constraints inherent to each problem considered (Stearns and Schmid-Hempel, 1987; Parker and Smith, 1990; Alexander, 1996).

One kind of biological problem, however, seems to be exempt from some of these objections: object manufacture and use. Unlike biological traits, objects manufactured by animals serve one or a very small set of well-defined or definable functions (Hansell and Ruxton, 2008; Shumaker et al., 2011). Manufacturing objects commands effort. Some objects even require an investment for a reward that appears much later (Finn et al., 2009). The existence of investments made in reshaping external objects, which the animal can chose not to make, suggests that object manufacture behavior is functionally important. Additionally, most objects are made solely using external materials which are themselves not under natural or co-evolutionary selection and can be chosen by the user for their functional properties. This makes the problem significantly simpler by limiting natural selection to the cognitive processes and morphology that supports object manufacture and use. Therefore, it is possible that animals can evolve, and indeed inherit, behavioral processes or routines that optimize the objects they manufacture.

Finally, the problem of teleology, here the intentional ‘goal-directed’ behavior of the animal, does not apply to inherited optimization processes which arise through natural or sexual selection. In these cases, the animal does not need to ‘intend’ to optimize the object. A facile and hard to test claim made sometimes is that object manufacture and its optimization ought to involve cognitive ‘insight’ or ‘innovation’ on the part of the animal. Such position can be seen as tantamount to claiming that the animal demonstrates teleological or conscious goal-directed action. While such cognitive capacity cannot be excluded *a priori*, we defend the idea that it is neither parsimonious nor a necessity for explaining object manufacture, use and optimization in animals, including insects.

## Heuristics or Not?

What are the search processes for finding the optima of objective functions? For optimization problems where the objective function is not predefined or is large, there are three broad search methods: (1) those that stop after a set number of steps – finitely terminating searches, (2) convergent methods that search iteratively and stop when objective function value converges, and (3) heuristic methods which do neither, but rather provide a ‘recipe’-like search method that is good at finding approximate solutions under certain circumstances.

A biological example of a finite terminating search in biology is a ‘best-of-*n*’ strategy for mate finding in which mate search is stopped after encounters with *N* males and the best male is chosen (Janetos, 1980; Dombrovsky and Perrin, 1994) or honey bees scouting a finite and set number of nest building sites and returning to the best of those sites (Seeley and Buhrman, 2001). In an object manufacture context, this would mean that the animal makes only *N* changes to the object and return to the object design that was the best among those *N* alternatives. An example of an iterative-convergent strategy would be the process by which mole crickets gradually improve the acoustic resonance of their singing burrow using sensory feedback (Bennet-Clark, 1987). In such a strategy, the mole cricket randomly changes different architectural features of the burrow, and continues only with those changes that make the burrow louder. Here, the important part is that the animal is monitoring the functional output rather than the architectural features of the structure it is building. An example of a heuristic search is baffle optimization in tree crickets which is guided by three rules that lead to an optimal baffle without a need for sensory feedback that monitors the cricket’s loudness (Mhatre et al., 2017). Baffles, much like mole cricket burrows, are acoustic aids that allow tree crickets to increase the loudness of their mating call. To make a baffle, a tree cricket must make a hole in a leaf and must sing from within this hole. In effect, there are three important features to a baffle, leaf size, hole size, and hole position. Three rules are sufficient to acoustically optimize the baffle, (1) pick the largest leaf, (2) make a hole the size of its wings, and (3) place the hole at the center of the leaf (Mhatre et al., 2017). Here, the important and noteworthy distinction resides in the fact that following a heuristic program does not require the animal to evaluate the functional output of the object being manufactured. The cricket does not evaluate the increase in song loudness. The cricket must instead evaluate the structural features, e.g., leaf size, hole size, and position, of the object it is manufacturing. The heuristic process encodes the optimal features of the manufactured object.

These optimization strategies are also not necessarily permanent. Animals, including small brained insects, can use learning, or indeed in some cases rule-abstraction (Avarguès-Weber et al., 2012) to transition from a convergent strategy to one that is heuristic. To explain how such a transition might function, we can use a purely hypothetical example: a hypothetical cricket that like the mole-cricket makes burrows to amplify its sound may make several burrows in its lifetime. It optimizes each burrow by evaluating its sound output. However, this cricket may, through experience, learn optimal burrow dimensions. In such a case, we expect that the starting dimensions of new burrows built by this male cricket would be closer and closer to the optimal burrow size indicating learning. Rule abstraction has indeed been observed in insects (Giurfa et al., 2001; Avarguès-Weber et al., 2012; Avarguès-Weber and Giurfa, 2013) but is much harder to establish, and often requires cleverly designed experiments. To provide an entirely hypothetical example here, we use a hypothetical cricket that makes a baffle similar to that made by tree-crickets. In the rule abstraction case, the cricket may make test baffles in two or more leaves and develop an abstract understanding that the larger leaf is always louder. In subsequent attempts at baffle manufacture, this cricket would chose the larger leaf to make the baffle. The best-test of true rule abstraction in this scenario would be to offer this hypothetical cricket the largest leaf it has previously encountered and one even larger than it. We expect that if this hypothetical cricket has abstracted the rule, it would always chose the larger leaf. However, one that has learned through experience and developed a preference for a particular leaf size, would be more likely to build in the smaller, but previously encountered leaf.

The tree cricket heuristic discussed here has one rule per problem dimension, i.e., a separate rule for every decision that has to be made. There are other, more generalized heuristics, such as the well-known genetic or evolutionary algorithms, the take-the-best algorithm, the diffusion model, and insect-inspired algorithms based on ant colony optimization (Bonabeau et al., 2000; Hutchinson and Gigerenzer, 2005; Cormen et al., 2009; Marshall et al., 2009). These algorithms use a smaller set of rules, and seek to be independent from the problem dimensions. Such heuristics have been developed to solve problems in optimization which cannot be solved analytically in a reasonable length of time. For high dimensional problems, these are the only available methods for optimization that can be accomplished at reasonable speed. While they are certainly faster, we can think of no *a priori* reason to believe that they will outperform iterative-convergent methods in accuracy. Given the diversity of these heuristics, we suggest that each heuristic must be carefully considered for the optimization problem at hand before such a decision can be made as to what optimization method, or methods, are at work and require testing. This decision is part of the research questions emerging in our own search to understand problem-solving in animals. More problematically, some of these methods need to sample the objective function during the search. We believe heuristics that require a sampling of the objective function should really be considered to be a subset of iterative-convergent processes, but those with specific rules that direct how the objective function should be sampled. Such heuristics essentially enhance the search process as compared to a random walk.

In optimization theory, the conventional wisdom is that iterative-convergent methods are more fine-grained and accurate at reaching optima and that non-convergent methods are essentially compromises that necessarily involve a lack of accuracy (Tversky and Kahneman, 1974). Within the context of optimization theory, finite terminating methods were specifically developed to save time, and heuristics were developed to improve search efficiency whether through saving time or through reducing computational and memory demands. Yet both methods are thought to sacrifice accuracy. Recently, however, we were able to show in a biological object-use system that a heuristic optimization method outlined for baffling-making tree-crickets would always outperform an iterative method in accuracy because the nature of the object being made disallows the animal from ‘editing’ the object (Mhatre et al., 2017). Here, we will consider other animal object-use systems and delineate (1) the conditions under which heuristic optimization can perform better not just in speed but also in accuracy and (2) those under which the more fine-grained iterative-convergent optimization methods would prevail.

## Object Properties and Manufacturing Techniques: Editing Objects

Iterative-convergent optimization requires that the optimized object be made stage by stage, and that the objective function be measured after each stage of manufacture. If the animal has ‘traveled up’ the objective function it is expected to continue in that direction, and it must be able to reverse direction if it has ‘traveled down.’ This is likely to imply, accepting process reversibility, that the animal must be able to undo changes it finds to be detrimental to object function. Such ‘editing’ may not be possible for all objects.

Some objects are made by a subtractive process, where a part of the object is removed to enable function. These objects are difficult to edit. Re-joining a removed part usually requires new manufacture techniques such as gluing (weaver ants and their larvae), stitching (tailor birds), or lashing with fibers (bagworm moth larvae). Such techniques may be unavailable to the animal, or be feasible only at some stages of manufacture. Tree cricket baffles, which serve the function of enhancing sound production, are made by cutting a hole in a leaf through which the animal sings (**Figure 1**). The objective function value of a particular baffle design is only meaningful with a completed hole. Once the baffle hole is made, it cannot be erased or ‘moved’ using the manufacture processes available to the tree crickets. Such editing would require that the crickets glue leaf pieces back, or weave a sheet across the hole, neither of which is an option for them. The crickets can only make a new hole if a current hole is acoustically suboptimal. In addition to the increased cost of manufacture, multiple holes in a single baffle leaf does compromise the acoustic advantage offered by the baffle (Mhatre et al., 2017). Thus, progressive optimization, while possible in baffle manufacture, is likely to be suboptimal compared to a relatively accurate heuristic if, as in this case, process reversibility is not afforded.

Even with additive processes, considerations of structural stability and the ability of animals to manipulate the required materials appropriately will limit how much a completed object can be edited. Adding or removing materials is only possible if the existing structure remains mechanically stable after the change, a problem akin to structural engineering. Additionally, the animal must be capable of making the required changes; for instance, it may be difficult to remove materials that harden after construction. The concept of irreversibility in the manufacturing process plays therefore a role in the domain of opportunities available and thus the evolution of behavioral strategies. Termite nests are designed to balance the dual needs of thermal regulation and gas exchange (Korb, 2003). Their architecture depends strongly on the environmental conditions (Korb, 2007, 2011; Hansell, 2007); savanna mounds have thinner walls and a complex external structure. Forest mounds have thicker walls, and a simple dome-like structure. When the environmental conditions of mounds were experimentally swapped, termites added complex external structures to the domed forest mounds, but did not remove pre-existing structures in the complex savanna mounds (Korb and Linsenmair, 1998).

## Organismal Properties: Sensory Systems and Memory Limitations

An iterative-convergent search method requires that the animal senses some stimulus that accurately reflects the objective function being optimized. Thus, the limitations of a sensory system, or the integrated detection envelope of sensory systems working together, can constrain the accuracy of optimization. For instance, a sensory system with low resolution in intensity coding would impair the detection of changes in the objective function, effectively equivalent to smoothing the objective function. This would result in the animal failing to identify optima altogether. Another example is based on the Weber–Fechner psychophysical law that applies to most sensory systems. The Weber–Fechner law predicts that the smallest perceivable change in a stimulus is proportional to the stimulus magnitude (Kingdom and Prins, 2016). Thus, when the stimulus intensity is already high, only a sufficiently large change in that stimulus is detectable. If and when seeking optimality, a sensory system needs to be able to detect small changes in the objective function, especially as the animal approach optimal values. Thus, an iterative-convergent search method may converge towards the optimal value, yet lose its resolving power whilst doing so, and only attain a sub-optimal value. An elegant example of such sub-optimal performance was recently shown in the case of the bat-bromeliad co-evolutionary dyad (Nachev et al., 2017). Bats were not able to discriminate between higher volumes or even concentrations of nectar, leading to an evolutionary persistence of plants with low quality nectars (Nachev et al., 2017).

In terms of object manufacture, an excellent example are mole crickets making specialized burrows that act as resonators and increase the intensity of their mating calls (Bennet-Clark, 1987). Based on the gradual improvement observed in burrow resonance, mole crickets are thought to be using an iterative-convergent search method to find optimal burrow dimension and geometry (Bennet-Clark, 1987). Three possibilities are suggested for the possible sensory cues used by the cricket; sound frequency, sound amplitude, and/or perhaps cuticular strain sensors that somehow monitor power output (Daws et al., 1996). Whatever the sensory mechanism used, a closer look at the burrow acoustics suggests that while burrow acoustics improve dramatically in loudness, they do not reach optimal tuning or loudness (Daws et al., 1996). The reason invoked is that sensory systems are not capable of, owing to Weber–Fechner law, reliably coding small changes in large stimuli.

In contrast, using a heuristic method with three simple rules would allow tree crickets to make an acoustically optimal baffle (Mhatre et al., 2017). So, why don’t mole crickets use a heuristic method? There may be several reasons for this, an important one is that it may not be possible to abstract the objective function for a burrow’s efficiency into a simple set of rules. The objective function of baffles has only three dimensions, is relatively smooth, and the optimization procedure can be coded by a rule per problem dimension as mentioned before (Mhatre et al., 2017). The mole cricket burrow optimization problem has a higher dimensionality since the value of several independent architectural features have to be determined, such as bulb length, bulb diameter, horn length, horn diameter, horn throat/constriction diameter, exit tunnel diameter, and excitation frequency (Daws et al., 1996). A specialized heuristic, such as the one used by tree crickets, that captures the optimal position on specific objective function will have at least one rule per dimension. Notably, if an identified parameter makes no difference to the objective function, the dimensionality of the problem is effectively reduced. On the other hand, if the shape of the objective function is complicated, more rules may be required. For instance, a conditional rule can exist that changes the rule for one parameter dimension depending on the value of another dimension. [For an arbitrary function *y* = f(*x*) if parameter *x* lies between 0 and 1, then choose the largest *y*, else if *x* is greater than 1, then choose the smallest *y*]. For a neural system adapted to handle several sensory modalities, encoding a large number of rules will run into the limit of the animal’s memory capacity. For instance, for the mole cricket burrow based on identified, but perhaps not complete, architectural features, we expect that at least seven rules are required. It is also expected that an iterative-convergent method will be computationally lighter and more efficient as the number of rules grow. Thus, iterative processes which are conventionally considered ‘higher cognition’ may actually be a strategy for reducing the rules that must be remembered and followed, thus minimizing computational and memory demands (McFarland, 1991).

Do sensory systems play no role in heuristic optimization? Indeed, they do; the heuristic decision making process is usually supported by some sensory information. For instance, in the tree cricket case, a larger leaf is chosen, a decision that requires information about leaf size. Additionally, tree crickets also size the hole with respect to their own wing size, and center it within the leaf. This process requires sensory information which enables them to size their wings and the hole and to find the leaf center. The important distinction between this heuristic and an iterative process, however, is that the cricket does not sense the functional output of the baffle (loudness), but rather its architectural features. Using the sensory system, however, means that errors can arise even in heuristic optimization. In the tree cricket system, errors have been observed in centring the baffle hole (Mhatre et al., 2017). In general, for the heuristic to outperform or equal iterative optimization, the performance-cost due to errors in these heuristic decisions must be lower than errors accrued from estimating the objective function stimulus directly.

## Organismal Properties: Collective Behavior

Insects are fairly unique among animals; through collective behavior, they can make objects that are made out of their own bodies. Remarkably, ants, bees, and wasps are deemed to make as many as 18 different kinds of self-assemblages (Anderson et al., 2002). A few examples encompass bridges that help them traverse gaps (Reid et al., 2015), rafts that enable them to survive in flood plains (Mlot et al., 2011), force generating clamps to hold the edges of a leaf together to sew them into a brood tent (Holldobler and Wilson, 1983), and anti-predatory ‘ovens’ which bees make by ‘balling’ around hornets to kill them by overheating (Ono et al., 1995).

Despite their difference from other objects we have considered so far, self-assemblages are also likely to be optimized. Since the assemblages do not use external objects or materials, selection remains confined to the insect’s cognition and morphology. Additionally, the assemblages serve crucial functions and are unlikely to be ‘spandrels.’ There is evidence for optimization in features such as a balancing of cost-benefit ratios in ant bridges (Reid et al., 2015; Graham et al., 2017), and very small tolerances for the temperatures achieved by the bee ovens which would kill the bees themselves if it increased by ∼2–4°C (Ono et al., 1995).

For collective structures, it is known that individuals change behavior and make decisions at speeds that preclude their being directed by a co-ordinating or leading individual (Couzin, 2007, 2009). Thus, there is no individual or entity that coordinates and monitors the performance of the object. Rather, the overall structure emerges from the decisions made by each individual based on simple rules which respond only to local information, i.e., via a heuristic (Anderson, 2002; Couzin, 2009).

Insects do make other types of objects through collective behavior, most notably the impressive habitations of the social insects – termite mounds, ant and wasp nests, and bees hives. These structures typically tend to be multifunctional and must fulfill the multiple demands made on habitations: providing protection from the external elements, against pathogens, parasites, and predators, also ensuring good climate control and ventilation, and fluid transport of goods and individuals within the nest. Thus, the objective function of these structures is likely to be more complicated and to summate these properties in a weighted fashion. Given the complexity of these structures, their size and the fact that they often grow continuously, it is also difficult to find the parameters that adequately describe the input space of these objects. Despite these difficulties, there has been some remarkable work recently in studies of collective building (Karsai and Wenzel, 2000; Perna et al., 2008a,b; King et al., 2015; Khuong et al., 2016) and the optimization of collectively built structures has been closely considered (Perna et al., 2012).

Given that there is no ‘co-ordinating’ individual, how is construction regulated in collectively built structures? As we understand it today, the main mechanisms that guide building are (1) stigmergy, i.e., insects interacting with the structure and (2) direct interactions between the insects themselves (Downing and Jeanne, 1988; Jeanne, 1996; Theraulaz and Bonabeau, 1999; Anderson, 2002). In stigmergic building, the construction behavior of the insect is directed by the structure or some of its features, i.e., the physical object it encounters or some chemical cue within this object can then drive its behavior. For instance, ants building a nest are more likely to deposit a pellet of building material in response to previously deposited pellet which has a high concentration of a pheromone, than next to one with a lower concentration (Khuong et al., 2016), whereas wasps may determine where to build the next cell based on the number of hexagonal sides free on the edge of the current structure (Karsai and Penzes, 1993). Direct interactions may regulate building, in particular in terms of nest size. It is suggested that population density determines the size and to an extent the structure of some ant nests (Franks et al., 1992; Buhl et al., 2004).

Is it possible for insects building structures collectively, whether with their own bodies or using other materials, to also use a different iterative-convergent method for optimizing this structure? The generation of an object by a heuristic approach does not necessarily preclude iterative-convergent optimization. In effect, as long as the object can be changed progressively, the optimization process can be separate from the construction process. However, the lack of a ‘co-ordinating’ individual does seem to prevent iterative-convergent optimization since there would be no examination of the global objective function and subsequent directing of behavior. Another possibility, however, is that each individual may sample the objective function, or a section of it, and modulate its building behavior accordingly (Perna et al., 2012). The difficulty in this scenario is that the structure being optimized is usually significantly larger than the insects building it and this limits their perceptual ability (Perna et al., 2012). However, if there were a stimulus that reflected the objective function, which was relatively homogenous within the structure, this might be a viable possibility. A simple example would be that the builders could monitor the temperature, CO_2_ or air-flow inside the nest, if these were relatively gradient free. However, typical nests are structured to generate gradients in these very features and these gradients are exploited to generate air-flows which ventilate, and redistribute heat within the nest (Korb, 2003; King et al., 2015). Another possibility is that the builders use sampling methods to estimate these quantities, as they have been shown to use to estimate nest size (Mallon and Franks, 2000; Mugford, 2001). However, the gradients within the structure are systematic (King et al., 2015), and even the structure of the nest itself is topologically systematic (Perna et al., 2008b). Therefore, simple random-walk based sampling methods would be insufficient and sampling within such structures would likely require an internal ‘map’ of the nest and spatial awareness. Cues such as nest temperature, humidity, airflow direction do modify insect building behavior (Korb and Linsenmair, 1998; Bollazzi and Roces, 2007, 2010b), but they are more likely to guide the modification of structures rather than the initial construction. We tackle this issue more completely in a later section.

In self-assemblages, the issue of information acquisition seems somewhat clearer. It is likely that the insect which is participating in the structure is likely to have access to only very local information and cannot access the global efficiency of the structure (Anderson, 2002; Anderson et al., 2002). For instance, in ant rafts, individual ants assemble to make a structure that floats because it is both buoyant and water repellent. (Mlot et al., 2011). While the rafts are made well enough to prevent even the ants on the bottom from drowning, the ants on the edges, bottom and in the center of the raft support different weights and have different oxygen supplies available to them (Mlot et al., 2011; Foster et al., 2014; Tennenbaum et al., 2016). Even where the behavior is purely mechanical, relying on some simple homogenous bulk quality such as the stiffness of the aggregate (Tennenbaum et al., 2016), such as in bridges or ropes, the forces experienced by animals at the edges and boundaries, will be different from those in the center, suggesting that local cues will differ from global cues preventing iterative-convergent optimization aimed at individual ants (Anderson, 2002). In general, we surmise that collective structures built using heuristic techniques are probably optimized in a similar fashion.

## Problem Properties: Changing Objective Functions

All the cases we have considered so far have static objective functions, i.e., the efficiency of an object of a particular design remains constant. In the real world, however, the efficiency of a particular object design may change as the object interacts with a changing environment, such as the changes in a nest’s efficiency as the light-shade regime changes or with changes in temperature, or in rainfall and humidity. Objective functions could also change when the object interacts with other organisms, for instance, traps. This could happen either through a slow evolutionary process such as an arms race between the trap maker and prey, or through faster processes such as the species composition and number of organisms the trap interacts with changes seasonally. Strictly speaking, environmental or organismal variables are not characteristics of the object, and hence cannot be incorporated as a dimension of the object’s state space. From the biological standpoint, however, optimization would require the animal to adapt the object and change its design to suit the changed conditions.

Invertebrate prey-capture traps come in two broad categories of design, pitfall traps or a web-based trap (Hansell, 2007; Scharf et al., 2011). Trap building involves structural considerations, for instance spider-webs need to be robust to environmental damage (Cranford et al., 2012; Sensenig et al., 2012; Qin et al., 2015), and functionally, these constructed objects may be used for other purposes such as mating rituals (Vibert et al., 2016). Their primary purpose, however, is food acquisition. Spider webs are complex structures with a wide variety of designs ranging from the commonly encountered two dimensional orb webs to the rarer three dimensional cobwebs of black widow spiders (Blackledge et al., 2009). It would be challenging to create a single and complete analytical framework to examine the entire range of web designs. Nonetheless, several authors have identified three functional features that are crucial to understanding trap efficiency: the ability to intercept, stop, and retain prey (Eberhard, 1990; Blackledge et al., 2011). To intercept prey, the traps must efficiently cover their capture area with silk, and make a web of appropriate mesh size. This web should either be relatively inconspicuous to prey or actually be attractive to prey. Next, to stop prey, webs must efficiently dissipate the kinetic energy imparted at prey impact without breaking or bouncing the prey off the web. This problem is largely addressed through different types of silk extruded by the spiders. Finally, the web must retain the prey, either by adhesion or by entanglement, another problem usually solved by using distinct silk types and, occasionally, structures such as ladders. It is known that different web designs have different efficiencies for each of these processes, and that spiders change their trap structure in response to their prey capture rate and nutritional status. Thus far, however, given the complexity of the problem much of the work addresses only a few features of spider web efficiency at a time (Eberhard, 1990; Blackledge and Zevenbergen, 2007; Zevenbergen et al., 2008; Blackledge et al., 2011; Blamires et al., 2016). Pitfall traps are simpler than webs. Among the most familiar are the pits of antlion larvae, which are conical depressions in loose sandy soil with the antlion hidden near the cone’s apex. The main features of the pit are its location, width, the slope of the walls and the particle size of the soil, and these features together determine the size of the prey captured and the likelihood that it will slip down the pit slope (Devetak et al., 2005; Fertin and Casas, 2006). Remarkably, the slope of the trap is optimized so as to be on the verge of the critical point of stability of the particular sand granularity, where a slight disturbance is poised to generate an avalanche leading the prey to the ambushing predator (Fertin and Casas, 2006). With pitfall traps, at least in some cases, trap size and structure appear to be also optimized for certain prey species (Devetak et al., 2005; Barkae et al., 2012).

How might traps be optimized given that the objective function of a particular design might change over its lifetime? The main theory covering optimization in this context is optimal foraging theory and there is some evidence that insects have the behavioral flexibility to optimize their foraging strategy (Scharf et al., 2011). If the approach to optimization remains purely heuristic, then the animal must switch between different rule sets for distinct functions. In addition, the animal has to sense some hallmark stimulus that indicates the transition from one objective function to another and chose the appropriate objective function for the transition. This leads to a problem that has been noted before: a large number of rules would have to be encoded into the heuristic (McFarland, 1991). In this situation an iterative convergent strategy might perform better. Indeed it has been reported that trap builders either evaluate trap efficiency directly, or through their own nutritional status, and use this imperfect information to guide trap modifications such as changing its size, shape, components, or location (Scharf et al., 2011). Such a process is suggestive of an optimization strategy that is iterative, rather than based on a bank of heuristic routines. However, both web or pitfall traps tend to have low and highly variable capture rates, whereby some traps catch nothing over several days (Edwards et al., 2009). This unpredictability is at the heart of the question of optimization, and makes it difficult to accurately assess trap efficiency (Blackledge et al., 2011; Scharf et al., 2011). Thus, even an iterative strategy may not be able to approach optimal design, and achieve the theoretically optimal foraging strategy. Interestingly, exit strategies exist; as traps are likely to be abandoned following trap damage, parasitic invasion, or competition, none of which relate to trap efficiency itself (Blackledge et al., 2011). Thus, trap or web abandonment may be indicative of boundary conditions of the objective function, or neighboring objective functions, and help test decision mechanisms and the logic of state-dependant transitions between strategies.

Unlike variations in prey distributions in time and space, environmental variations may be considered more predictable since they are often brought about by circadian or seasonal rhythms. At the local scale of the ecological niche of small animals, uncertainty in both trophic and abiotic factors prevails, constituting part of the challenge in the search for optimality. Nest building insects seem to have developed both active and passive mechanisms for dealing with variation and uncertainty (Jones and Oldroyd, 2006). The most common passive adaptation for dealing with temperature variations is nest insulation which helps maintain a steady internal nest temperature. This tolerance is achieved by several mechanisms, such as multi-layered insulation within the nest structure as observed in stingless bee species (Roubik, 2006), or by orienting the nest with respect to the sun in order to harness solar heating as observed in magnetic termite mounds (Jacklyn, 2010). As these variations are of the type that can be anticipated, the nest can be structured, and actively modified, with available manufacture methods. For instance, some ants regulate nest temperature and humidity by plugging and unplugging air vents which are made only to regulate temperature and humidity and do not function as entrances (Franks, 1989; Bollazzi and Roces, 2007, 2010a,b). Another common mechanism for dealing with environmental changes, such as seasonal changes is simply to make short-lived nests and abandon them for new nests developed for the newer conditions, a behavior seen in ants that build different winter and summer nests (Ofer, 1970). Bees and wasps are well known to heat up their nest by using metabolic heat generated by rapidly contracting their flight muscles (Kronenberg and Heller, 1982). Inversely, bees can cool their nest down, whereby water deposited on the nest surface evaporates and serves to lower nest temperature (Ishay and Barenholz-Paniry, 1995). Arguably, the most intriguing passive thermoregulatory mechanism uses thermoelectric material properties; the silk of pupal cases in the hornet appears to accumulate electric charge under hot conditions, and releases it during cold conditions and helps maintain pupal temperature (Ishay and Barenholz-Paniry, 1995).

Given the wide range of mechanisms, it is difficult at the moment to make a single argument for whether the adjustment of optimization points is carried out heuristically, or using an iterative-convergent method, or a mix thereof. A few different possibilities exist: where passive insulation is important, no new behavior is required; where new structures are built, they may be built using a different set of optimized rules with the cue for the shift being a seasonal rather than nest-based cue, such as day–night length. This would connote a heuristic process of re-optimization. Where the nest is modified in some fashion, however, the insects must receive some nest-efficiency cue that initiates the re-optimization procedure. Whilst such a cue may be sensed by individual insects, and may result in a new emergent collective behavior that seeks a novel optimum, it may perhaps never reach this optimum, but might nonetheless adapt to a novel state space. Such a system dynamics view may be useful to experimentally identify key cues that prompt such transitions. Quite certainly non-linear, such transitions may be the key to the presence of adaptive fast heuristics. However, as we have discussed in the context of collective construction, cues within the nest are variable, and more importantly vary systematically. As individual insects are likely not to have access to a global measure of efficiency of the nest, it may be useful to hypothesize that local conditions ought to provide sufficient information that locally engages many individuals into the proper heuristics. Of course, these considerations are not limited to insects but also encompass vertebrates that build collectively, such as the African social weavers (Van Dijk et al., 2013). One of the ways that nesting insects solve this problem is to monitor temperature where it is most crucial. Nest temperature has a large effect on brood development. Thus, local monitoring of the brood chamber and, when necessary, moving brood to other parts of the nest where temperatures are more favorable is an effective strategy (Jones and Oldroyd, 2006). Another possibility is that the variation in nest temperature may not be a significant factor in nest optimization. Indeed, some modeling studies suggest that variance in the sensitivity to temperature in nest building insects may actually be important in stabilizing the temperature within the nest (Myerscough and Oldroyd, 2004; Graham et al., 2006; Jones and Oldroyd, 2006).

## Conclusion: Integrating Models and Behavior

The question of how seemingly “complex” behaviors such as object optimization are organized in the so-called “simple” organisms can benefit from a careful disssection of the physical dimensions of the problem and the different approaches available for solving the problem. We suggest here that a basic approach, such as heuristics, can help explain behavioral adaptations without the requirement of large neuronal processing power. This proposition is naturally complementary, and not exclusive, to other solutions employing such brain power. The particulars of object use and optimization is only one of many realms of application, much of it remains to be explored in terms of heuristic optimization. In effect, our analysis of object use in insects has, by itself, implications for how we can gain a more complete understanding of the living world. In particular, there is much to gain by examining the organizational patterns that connect organisms with the physical aspects of their ecology. With this respect, predictive models anchored in the physics of the manufactured objects are needed that can identify objective functions and their key parameters, capture boundary conditions and characterize feasible domains. Such models can directly help formulate testable hypotheses and test behavioral decisions and their consequences (e.g., Perna et al., 2008a,b). In particular, the power of analytical methods traditionally used in engineering, such as finite element modeling and analysis, are increasingly applicable to heterogenous and dynamic biological structures. Used in conjunction with high-resolution X-ray tomography and 3D printing, much insight could be gained from modeling and experimental approaches.

In its own and perhaps small way, this heuristic approach challenges the overused, poorly supported and dysfunctional metaphysical category “simple.” Celebrating the power of observation, when considered for long enough, insight ensues and nothing becomes simple.

## Author Contributions

NM performed the original research and data analysis, and led the MS design and writing. NM and DR conceived of the research and wrote the manuscript.

## Conflict of Interest Statement

The authors declare that the research was conducted in the absence of any commercial or financial relationships that could be construed as a potential conflict of interest. The reviewer AA-W and handling Editor declared their shared affiliation.
